# Supplementary material for: Home-based exercise for people living with frailty and chronic kidney disease: A mixed-methods pilot randomised controlled trial
Source: PLoS One. 2021 Jul 1;16(7):e0251652. doi: 10.1371/journal.pone.0251652 (PMC8248609; doi:10.1371/journal.pone.0251652)
Supplement: S1 Table — (DOCX) [file pone.0251652.s001.docx]

|  | Total | Percentage |
| --- | --- | --- |
| CFS score | 257 | 57.4 |
| Participation considered unsafe | 70 | 15.6 |
| Unable to mobilise independently | 21 | 4.7 |
| CKD stage | 20 | 4.5 |
| Insufficient understanding of English language | 15 | 3.3 |
| Anticipated to commence dialysis or receive a renal transplant within the next 3 months | 12 | 2.7 |
| Persistent uncontrolled hypertension | 11 | 2.5 |
| Recently enrolled in a structured exercise programme | 11 | 2.5 |
| Unable to give informed consent | 11 | 2.5 |
| Receiving palliative care for advanced terminal cancer | 5 | 1.1 |
| Registered blind | 5 | 1.1 |
| Unstable angina/recent myocardial infarction | 4 | 0.9 |
| Uncontrolled arrhythmia | 3 | 0.7 |
| Age | 1 | 0.2 |
| Current active enrolment in another interventional study | 1 | 0.2 |
| Mortality | 1 | 0.2 |

**S1 Table. Reasons for Ineligibility.**

CFS, Clinical Frailty Scale; CKD, Chronic Kidney Disease.
