# Supplementary material for: Home-based exercise for people living with frailty and chronic kidney disease: A mixed-methods pilot randomised controlled trial
Source: PLoS One. 2021 Jul 1;16(7):e0251652. doi: 10.1371/journal.pone.0251652 (PMC8248609; doi:10.1371/journal.pone.0251652)
Supplement: S2 Table — (DOCX) [file pone.0251652.s002.docx]

**S2 Table. Reported Reasons for Declining Study Participation.**

|  | Total | Percentage |
| --- | --- | --- |
| Study burden | 27 | 36 |
| Feeling unwell | 26 | 35 |
| Perceived ineligibility | 7 | 9 |
| Carer responsibilities | 6 | 8 |
| Travel | 5 | 7 |
| Time constraints | 3 | 4 |
