# Supplementary material for: Home-based exercise for people living with frailty and chronic kidney disease: A mixed-methods pilot randomised controlled trial
Source: PLoS One. 2021 Jul 1;16(7):e0251652. doi: 10.1371/journal.pone.0251652 (PMC8248609; doi:10.1371/journal.pone.0251652)
Supplement: S3 Table — (DOCX) [file pone.0251652.s003.docx]

**S3 Table. Qualitative Study: Participant Demographics and Clinical Characteristics.**

|  | Usual Care  (n=7) | Exercise  (n=8) |
| --- | --- | --- |
| Age, years, mean ±SD | 80.1 ±3.5 | 76.9 ±7.5 |
| Female, n (%) | 5 (71) | 3 (38) |
| Ethnicity  - White British, n (%) | 7 (100) | 8 (100) |
| Primary Renal Disease, n (%)  - Renovascular/Ischaemic  - Diabetic  - Cardio-renal  - Glomerulonephritis  - Obstructive Uropathy  - Other  - Unknown | 4 (57)  1 (14)  0 (0)  0 (0)  0 (0)  1 (14)  1 (14) | 4 (50)  0 (0)  1 (13)  0 (0)  2 (25)  1 (13)  0 (0) |
| CCI, mean ±SD | 3.7 ±1.0 | 3.9 ±1.4 |
| Medications, median (IQR) | 8.0 (9.0) | 7.0 (5.5) |
| Smoking History, n (%)  - Non-smoker  - Ex-smoker  - Current smoker | 4 (57)  3 (43)  0 (0) | 2 (25)  5 (63)  1 (13) |
| Living Circumstances, n (%)  - Alone  - With Family | 4 (57)  3 (43) | 5 (63)  3 (38) |
| Received Carer Support, n (%) | 0 (0) | 0 (0) |
| CFS Score, n (%)  - 4: Vulnerable  - 5: Mildly frail  - 6: Moderately frail | 3 (43)  0 (0)  4 (57) | 7 (88)  1 (13)  0 (0) |
| Frailty Phenotype  - Pre-Frail  - Frail | 5 (71)  2 (29) | 6 (75)  2 (25) |
| Fall Previous 6 Months, n (%) | 1 (0) | 0 (0) |
| BMI, kg/m^2^, mean ±SD | 30.1 ±6.9 | 28.1 ±6.6 |
| Blood Pressure, mean ±SD  - Systolic, mmHg  - Diastolic, mmHg | 141.9 ±8.4  70.9 ±9.0 | 142.0 ±21.1  72.1 ±9.6 |
| Laboratory Variables, mean ±SD  - Creatinine, μmol/L  - eGFR, ml/min/1.73m^2^  - Haemoglobin, g/L  - Albumin, g/L | 242.0 ±56.7  18.6 ±6.6  117.3 ±19.8  40.6 ±3.8 | 290.8 ±110.9  17.4 ±6.3  117.6 ±5.8  40.8 ±1.8 |

CCI, Charlson Comorbidity Index; BMI, Body Mass Index; eGFR, estimated Glomerular Filtration Rate.

Data presented as number (%), mean ± SD or median (IQR).
