# Supplementary material for: Home-based exercise for people living with frailty and chronic kidney disease: A mixed-methods pilot randomised controlled trial
Source: PLoS One. 2021 Jul 1;16(7):e0251652. doi: 10.1371/journal.pone.0251652 (PMC8248609; doi:10.1371/journal.pone.0251652)
Supplement: S4 Table — (DOCX) [file pone.0251652.s004.docx]

**S4 Table. Qualitative Study: Themes and Supporting Quotes.**

| Category | Themes | Sub-Themes and Supporting Quotes |
| --- | --- | --- |
| Trial | Decision to participate | Altruism: *“I don't know I just think 'hey, we're on this earth once and if we can do a bit of good and some people learn from it, then it's a bonus’.”* [Participant 24: age 77; male; pre-frail; exercise group]  Influence (family): *“I have a sister who is a retired district nurse. I have my youngest son who used to be an air medic in the RAF and they both said it would do me good.”* [Participant 17: age 72; male; frail; exercise group]  Influence (health professional): *“Well I don't think anybody else would have suggested it somehow, it's just I happened to see [Doctor] that day. Sometimes I see another man but he's learning so if he'd given it me I might have thought 'Mmm not sure'. But [Doctor], I trust [Doctor], whatever he says as far as I'm concerned that's it.”* [Participant 20: age 80; female; pre-frail; exercise group]  Influence (health professional): *“… I just had doubts because of my lack of mobility. So, I phoned up and they said no still come, so that's why I'm here.”* [Participant 6: age 80; female; pre-frail; usual care group]  Personal gain: *“Well I look at this way, if the doctor offers you to go on a study that may or may not help you, you have a choice and the thing about it is at my age I'm sick of being ill. And anything even if it’s only five per cent improvement in my life and my well-being then I felt that, you know? It was my obligation to me.”* [Participant 7: age 71; female; frail; exercise group] |
|  | Attitude to randomisation | Indifference: *“I wouldn’t have bothered, you know? I would’ve just carried on with my life as normal and then come in and done the test...”* [Participant 4: age 65; male; pre-frail; exercise group]  Rejection: *“I'm not sure on that one. There’s a possibility I would have come back to ask why? Why I wasn't good enough.”* [Participant 20: age 80; female; pre-frail; exercise group]  Disappointment: *“I was a bit disappointed when you sort of turned me down… Well I wanted to improve my ability to walk and anything else that would help me…”* [Participant 28: age 83; female; frail; usual care group] |
|  | Perception of frailty | Acceptance: *“That's the thing that intrigued me. That word... When I first decided to come on it. Because yes, I was frail and I was getting worse, even though I do the exercises that I do.”* [Participant 17: age 72; male; frail; exercise group]  Unease: *“You’d made me think ‘I was oh God’, you know? ‘Am I that frail?’ And I think I am. But on the other hand, I try not to think… I think, I feel like I wanted to fight it.”* [Participant 18: age 83; female; frail; usual care group]  Rejection: *“I thought that was a bit queer really, frail. I didn’t regard myself as frail. But anyway.”* [Participant 14: age 89; male; pre-frail; exercise group] |
|  | Experience of outcome assessments | Understanding: *“There has to be measures don’t there in anything? So, you know, questions have to be asked to be able… got to have somewhere when you start and when you finish and if there isn’t those sort of questions then you wouldn’t have learned anything.”* [Participant 4: age 65; male; pre-frail; exercise group]  Understanding: *“I thought they were very thorough and easy to understand and you explained things to me before I started doing them. No I was quite happy to fill the, all the forms in.”* [Participant 6: age 80; female; pre-frail; usual care group]  Acceptance: *“I knew there’d be a lot of questionnaires and things like that, because that’s the only way you find out about things.”* [Participant 28: age 83; female; frail; usual care group]  Safety: *“Yeah but thing about it is you don't want to make a fool of yourself but also you know when you have people what I call is catchers in case you know toppled over, that was good because it give me a little bit more confidence.”* [Participant 7: age 71; female; frail; exercise group]  Intrusive (privacy): *“Yes there was a couple but there was, it weren’t uncomfortable, it were like, ‘Really? Do you really need to know this?’.”* [Participant 7: age 71; female; frail; exercise group]  Intrusive (time): *“I got bored, I were ready for throwing them in the bin if I were honest. If there were a bin next to me I think you would have found them all shredded because it was just too much.”* [Participant 7: age 71; female; frail; exercise group] |
| Intervention | Challenges, doubts and fears | Challenges: *“I still…certainly the one in the chair pushing up, I still get a bit breathless with that but not as much as I used to.”* [Participant 17: age 72; male; frail; exercise group]  Challenges: *“I think the sitting down and the standing up etc. pushed me a little bit to the limit at times.”* [Participant 1: age 84; male; pre-frail; usual care group]  Doubts: *“I can only walk a certain distance and have to sit down and if there’s no seats… I can't walk and walk you know?… So, I'm snookered as they say.”* [Participant 21: age 81; female; pre-frail; usual care group]  Fears: *“This knee just went 'oh no we're not doing that'. It's kind of like whoa I’m getting a bit old now and I don't want new kneecaps or new hips or anything like that so I think I’d better pull out. Listen, to what me body tells you, your body says 'oh no no'.”* [Participant 24: age 77; male; pre-frail; exercise group]  Fears: *“I do know that if I over stress myself walking or anything, I get a lot of pain with my neuropathy and if I’ve walked quite some distance, I do get cramp.”* [Participant 6: age 80; female; pre-frail; usual care group] |
|  | Importance of staff attitude | Sympathy/empathy: *“But the thing about it is that she was very very helpful. She was sympathetic to my disability and that was lovely. That was something that I find is something that is very good when you get somebody that you've got a warmth to that is there to help you and not to… ‘well you need to do this and you need to do that’. She didn't. She was just very helpful.”* [Participant 7: age 71; female; frail; exercise group]  Patience: *“[Physiotherapist], that did it, was very patient with you and explained everything in very good detail so that you knew exactly how to do it and what to look for when you were doing it. And what, if you got to your limit, you know, she was like stop and rest and take your time and I found that very good.”* [Participant 17: age 72; male; frail; exercise group]  Individualisation: “…but after speaking to you and saying right well instead of doing tens drop that one down to five see how you go with that, in some cases that was the exact thing that I needed and that gave me a bit of get up and go, it didn't seem, it didn’t seem too strenuous.” [Participant 7: age 71; female; frail; exercise group]  Individualisation: “…if I said things you either said ‘no’ or ‘try this way’ it was like when I was doing the raises the leg raises and then doing the toe thing you told me to change them round because my legs wouldn't work to do that.” [Participant 20: age 80; female; pre-frail; exercise group]  Encouragement: *“But as I felt more confident in myself that’s when we talked on the phone that I decided to up it up a little bit.”* [Participant 7: age 71; female; frail; exercise group]  Patronisation: *“We were treated like, well I felt treated a bit like a little girl and I'm not a little girl. It's like some a teacher talking to a child type of thing.”* [Participant 20: age 80; female; pre-frail; exercise group]  Patronisation: *“…and I think, ‘no, that's written for geriatrics and I’m not quite a geriatric. I might be getting old but I’m not a geriatric yet’, you know?”* [Participant 24: age 77; male; pre-frail; exercise group] |
|  | Engaging with intervention materials | Contemplation and collaboration: *“I went away thinking about it. All the way home I was thinking. Yes right. This is what I've got to do. I was fine with that.”* [Participant 20: age 80; female; pre-frail; exercise group]  Willingness to practise: *“For me I found it [RPE], once I got used to it, I found it very easy to do it and it was pretty straightforward. But as I said at first, two or three times, it was a bit difficult to assess and I kept having to look back on the forms on your coloured chart to see that’s that one, that’s that one.”* [Participant 17: age 72; male; frail; exercise group]  Dismissing unfamiliar concepts: *“No. I just I've never ever worked with anything like that [RPE] at all, you know? How hard was it from one to 10? Come on, you know? It's an alien concept to me, you know?”* [Participant 24: age 77; male; pre-frail; exercise group] |
|  | Motivations to (or not to) exercise | Personal goals: *“Well I thought it might make me feel better in myself. I feel a bit down so I thought maybe exercise, I know I walk but I can't walk fast, you know? So, I thought if I do exercise it might make me go get my mojo back.”* [Participant 13: age 77; female; pre-frail; usual care group]  Personal goals: *“I used to be very active, especially at work and this like 10 years since I retired I've gradually gone down and I’ve been trying to get it back up again.”* [Participant 17: age 72; male; frail; exercise group]  Personal goals: *“I know that I’m getting older, but I don't want to be decrepit.”* [Participant 28: age 83; female; frail; usual care group]  Personal goals: *“Well I wanted to improve my ability to walk and anything else that would help me.”* [Participant 28: age 83; female; frail; usual care group]  Self-determination: *“I'm going to try and keep doing these exercises as long as I can. Just keep going.”* [Participant 16: age 78; female; pre-frail; exercise group]  Self-determination: *“Well I wanted to progress as far as I could. I wasn’t going to quit. There was no way I would quit part way through it.”* [Participant 14: age 89; male; pre-frail; exercise group]  Resilience: *“Personally though, I've got kidney disease, but I never think about it. I didn't regard it as a handicap. I’ve done whatever I did before, despite the kidney disease. It’s never held me back at all. You know, you’ve got to just carry on… Keep going… No, the kidney disease has never held me back.”* [Participant 14: age 89; male; pre-frail; exercise group]  Resilience: *“Trying to keep going, yeah, there’s no point giving in.”* [Participant 28: age 83; female; frail; usual care group]  Personal responsibility: *“You can't complain about something if you signed up for it and you say you’re going to do it and then start moaning about it. It’s not on, is it?”* [Participant 24: age 77; male; pre-frail; exercise group]  Sense of achievement: *“Well it was achievement because at first I was ‘can I do this?’ but it was just nice when things were levelled up when five minutes became ten minutes walking. That and I had difficulty with exercise five, the press up one, but then you said, ‘well do five, see how you go with that’. And then again, I built up to ten and I've got that off perfect now, it really is perfect now. [Interviewer: “And how does that make you feel?”] Good, ‘cause I could do it without any pain in my arms and that was it. And it was absolutely brilliant yesterday.”* [Participant 20: age 80; female; pre-frail; exercise group]  Telephone calls with research team: *“… you know if somebody’s going to phone up to see whether you’ve done them or not, it makes you keep going.”* [Participant 16: age 78; female; pre-frail; exercise group]  Location: *“Personally, I’d rather go where there’s other people.”* [Participant 18: age 83; female; frail; usual care group]  Location: *“Personally I found it better being able to do it in the privacy of my own home.”* [Participant 17: age 72; male; frail; exercise group]  Location: *“I prefer doing them at home, yes. I can do it when you feel like you've got the energy to do it. If you go to a class sometimes you’re at that point of a day you’re not feeling too good. When you can do it at home in your own time. You get up and do it when you feel you've got the energy to do it.”* [Participant 16: age 78; female; pre-frail; exercise group]  Enjoyable: *“As you know I have a lot of problems with me joints and one or two of the exercises I've found quite difficult. But some of them I found quite enjoyable and also because of enjoyment I seem to find that it worked better.”* [Participant 7: age 71; female; frail; exercise group]  Unenjoyable: *“It wasn't a chore as such, it was something that I had to do.”* [Participant 24: age 77; male; pre-frail; exercise group]  Perceived ineffective: *“But I don’t know, I sat down and thought about it and I thought, ‘I really can't see how it's helping me as such’.”* [Participant 24: age 77; male; pre-frail; exercise group] |
|  | Improvements in physical health | Fitness: *“As I say, I found it very helpful and I'm certainly fitter than, I think I'm fitter, feel fitter than I did before, three months ago when I started it.”* [Participant 17: age 72; male; frail; exercise group]  Fitness: *“Well like I said I can reach into cupboards, I can go upstairs easier, I’m walking better. Yes, it's been a good thing.”* [Participant 14: age 89; male; pre-frail; exercise group]  Fitness: *“I used to get out of breath going upstairs and I don't now. It's made a big difference to that.”* [Participant 14: age 89; male; pre-frail; exercise group]  Balance: *“…my balance is a lot better. I walk straighter when I’m shopping.”* [Participant 20: age 80; female; pre-frail; exercise group]  Strength: *“I've found it very interesting. And it's helped me a lot, in getting my strength back up, ‘cause I'd got very weak.”* [Participant 17: age 72; male; frail; exercise group] |
|  | Improvements beyond the physical | Well-being: *“I’ve found it, I really found it very helpful, very very beneficial to my overall health. I think it’s made me more content with my life.”* [Participant 17: age 72; male; frail; exercise group]  Well-being: *“Funny enough after I felt, after I finished them and I go and sit down, I feel great, as if it’s lifted me a bit, you know? It’s like took years off, you know?”* [Participant 9: age 83; male; pre-frail; exercise group]  Energy: *“I feel not quite as knackered as I did before.”* [Participant 4: age 65; male; pre-frail; exercise group]  Confidence: *“But I think now because I've done them I'm not as afraid of walking as I was…”* [Participant 7: age 71; female; frail; exercise group]  Confidence: *“I feel better about myself and my confidence”* [Participant 20: age 80; female; pre-frail; exercise group] |
