# Supplementary material for: Home-based exercise for people living with frailty and chronic kidney disease: A mixed-methods pilot randomised controlled trial
Source: PLoS One. 2021 Jul 1;16(7):e0251652. doi: 10.1371/journal.pone.0251652 (PMC8248609; doi:10.1371/journal.pone.0251652)
Supplement: S2 File — (PDF) [file pone.0251652.s006.pdf]

The EX-FRAIL CKD Trial: a pilot mixed-methods randomised controlled trial of a home-based EXercise programme for pre-FRAIL and FRAIL, older adults with chronic kidney disease

Protocol Version: 0.6 (02/05/2018)

1. LAY SUMMARY

Frailty is very common in people with chronic kidney disease. It is linked with disability, poorer quality of life and an increased risk of death. Despite knowing this, we do not know what is the best way to care for those that are frail with chronic kidney disease.

Studies have demonstrated that exercise appears helpful for frail older people. However, frail people with chronic kidney disease have health problems that are different to older people with normal kidneys. Muscle wasting appears to be more severe as kidney failure worsens. Exercise may decrease the muscle wasting that occurs and improve the overall health and quality of life of frail patients with chronic kidney disease.

Most research studies of exercise programmes are done under close supervision outside a person's own home. They are difficult to set up as they are expensive and require a lot of time from busy staff. They may also be demanding for frail older people as they need to travel to take part in exercise. Research is needed to explore the benefits of a home-based exercise programme for frail older people with chronic kidney disease.

We propose a preliminary study to be conducted over 12-months that investigates whether it would be possible to perform a larger study that examines the benefits of a home-based exercise programme for frail older people with chronic kidney disease. We have received feedback on our study from members of a local kidney charity, a group of older patients with chronic kidney disease and members of the public. We have modified the study based upon this feedback.

In our study, those that are willing and eligible to take part will be divided into two groups. The first group will be taught by physiotherapists how to perform the exercise programme. They will be asked to perform the exercises at home three times a week for 12-weeks. The second group will not receive the exercise teaching. We will assess the physical ability and quality of life of participants in both groups at the beginning and end of the 12-weeks. We

will also ask some participants to take part in interviews that explore their experience of the study.

If we can successfully perform this preliminary study and participants find the study acceptable, we will use the information gathered to design a larger study that examines the benefits of a home-based exercise programme for frail older people with chronic kidney disease.

2. ABSTRACT

Frailty is a significant health-burden in the chronic kidney disease (CKD) population. Frailty is associated with progressive renal impairment with a prevalence of >60% in dialysis-dependent CKD patients, compared with a prevalence of 11% in the general older adult population.(1-3) Crucially, frailty is independently linked with adverse clinical outcomes in all stages of CKD, including an increased risk of worse quality of life, falls, hospitalisation and mortality.(1, 4-9) Despite this knowledge, evidence for specific management strategies for pre-frail and frail patients with CKD is lacking.

Studies have demonstrated that exercise training appears to be beneficial for frail older adults.(10-12) However, frail patients with CKD represent a sub-group of patients that are not directly comparable to the general older population.(13) This is a result of the various pathophysiological processes inherent to CKD, including the accumulation of uraemic toxins, an increased inflammatory burden, anabolic hormone dysregulation, metabolic acidosis and CKD-mineral bone disease.(13) Evidence has demonstrated that there is significant muscle wasting, a major contributor to physical frailty, prior to the commencement of dialysis.(14) Thus, there is a need for studies that specifically evaluate exercise programmes for those with pre-dialysis CKD. Prompt exercise rehabilitation may mitigate against this muscle wasting and limit the propagation from robustness to frailty. Finally, most exercise programmes used in studies involving participants with CKD have been performed in intensively supervised environments, conditions that are challenging to implement in clinical practice considering financial constraints and staffing limitations.(15, 16) Such exercise programmes also confer significant travel demands that may be onerous for pre-frail and frail individuals with CKD. Research is needed to evaluate more pragmatic home-based exercise programmes.

We propose a pilot mixed-methods randomised controlled trial of a home-based exercise programme for pre-frail and frail older adults with CKD to be conducted over a 12-month period. The main objective is to evaluate the feasibility of performing a larger definitive randomised controlled trial that investigates the effect of a home-based exercise

intervention on physical function and quality of life in pre-frail and frail older patients with CKD.

3. BACKGROUND AND STUDY RATIONALE

Frailty is a devastating complication of chronic kidney disease (CKD). Frailty is strongly associated with progressive renal impairment and is independently linked with adverse clinical outcomes in all stages of CKD, including an increased risk of worse quality of life, falls, hospitalisation and mortality.(1-9) Reviews of various exercise programmes for patients with CKD have determined that exercise training offers numerous health benefits.(10-12) However, studies to date have not targeted pre-frail and frail patients with CKD, a vulnerable patient group who potentially have the most to be gained from an individualised exercise rehabilitation programme. The James Lind Priority Setting Partnership has stated that research is required that investigates the impact of exercise and physical activity in preventing and managing frailty. Frailty is also considered a key theme in the ongoing NIHR highlight notice of 'Complex Health and Care Needs in Older People'. Furthermore, the UK Renal Research Strategy has highlighted the importance of research into the prevention of complications of CKD.

Studies have demonstrated that exercise training appears to be beneficial for frail older adults. However, frail patients with CKD represent a sub-group of patients that are not directly comparable to the general older population.(13) This is a result of the various pathophysiological processes inherent to CKD, including the accumulation of uraemic toxins, an increased inflammatory burden, anabolic hormone dysregulation, metabolic acidosis and CKD-mineral bone disease.(13) Evidence has demonstrated that there is significant muscle wasting, a major contributor to physical frailty, prior to the commencement of dialysis.(14) Thus, there is a need for studies that specifically evaluate exercise programmes for those with pre-dialysis CKD. Prompt exercise rehabilitation may mitigate against this muscle wasting and limit the propagation from robustness to frailty that is associated with progressive decline in renal function. Finally, most exercise programmes used in studies involving participants with CKD have been performed in intensively supervised environments, conditions that are challenging to implement in clinical practice considering financial constraints and staffing limitations.(15, 16) Such exercise programmes also confer significant travel demands that may be onerous for pre-frail and frail individuals with CKD.

Research is needed to evaluate a more pragmatic home-based exercise programme for this patient cohort.

We propose a pilot mixed-methods randomised controlled trial of a home-based exercise programme for pre-frail and frail older adults with CKD to be conducted over a 12-month period. The main objective is to evaluate the feasibility of performing a larger definitive randomised controlled trial that investigates the effect of a home-based exercise intervention on physical function and quality of life in pre-frail and frail older adults with CKD.

4. OBJECTIVES

The study's objectives are to:

- Assess the acceptability of randomisation within the EX-FRAIL CKD study to pre-frail and frail participants with CKD.
- Assess the acceptability of the EX-FRAIL CKD study outcome measures to pre-frail and frail participants with CKD.
- Assess the acceptability of the EX-FRAIL CKD exercise programme to pre-frail and frail participants with CKD.
- Assess the feasibility of pre-frail and frail participants with CKD adhering to the EX-FRAIL CKD exercise programme.
- Assess the feasibility of recruiting pre-frail and frail participants with CKD in the outpatient nephrology setting.
- Collect data that will inform the design of a larger definitive randomised controlled trial including estimation of sample size.

5. STUDY DESIGN

This is a pilot mixed-methods parallel randomised controlled trial of a home-based exercise programme for pre-frail and frail older adults with chronic kidney disease (CKD) that is to be conducted over a 12-month period. The main objective is to evaluate the feasibility of performing a larger definitive randomised controlled trial that investigates the effect of a home-based exercise intervention on physical function and quality of life in pre-frail and frail older patients with CKD.

All participants will be ≥65 years of age, will have CKD stage 4 or 5 and will be registered with the Department of Renal Medicine at Lancashire Teaching Hospitals NHS Foundation Trust (LTHTR). Participants will have been assessed as having a Clinical Frailty Scale (CFS) score ≥4.(17) Only those meeting the definitions for pre-frailty and frailty when assessed by the Fried Phenotype Criteria (FP) will be eligible for randomisation.(18) Those that are classified as robust despite a CFS score ≥4 will still receive advice on safe exercise practices but will not be eligible for randomisation and will be withdrawn from the study.

Eligible participants will be randomly assigned to an exercise (plus standard care) or standard care alone group. Baseline assessments will be performed at day 1. Participants allocated to exercise will receive education on the exercise programme and provided with an exercise instruction manual. They will perform the exercise programme over 12-weeks. Further study assessments will be performed at the end of week 12 for both groups.

Recruitment to a qualitative sub-study will begin once participants have completed the 12-week study assessments and will continue until a heterogeneous sample (based on age, gender and frailty status) has been achieved. A representative sample of participants from both study groups will be invited to participate in semi-structured, individual interviews exploring their experience of the trial and, where applicable, the intervention.

6. SELECTION OF STUDY POPULATION AND WITHDRAWAL CRITERIA

6.1. Screening

Consultant Nephrologists at LTHTR will assess eligibility of potential participants during their General Nephrology clinics. Consultant Nephrologists will use the CFS to screen for pre-frailty and frailty (score ≥4).(17) Potential participants will be provided with a participant information leaflet and asked to contact the research team if they are interested in participating in the study. If a potential participant is interested in discussing participation in the study, a further meeting will be arranged with a member of the research team at which the potential participant will have the opportunity to ask questions about their involvement in the study. If at the meeting the potential participant has read the participant information leaflet and reflected on their involvement in the study, the research team will obtain formal written consent. No study assessments or procedures will be performed prior to this. At this or a further study visit a formal frailty assessment using the FP criteria will be performed.(18) Only those meeting the definitions for pre-frailty and frailty will continue with further study assessments and will be eligible for randomisation. Those that are classified as robust by FP criteria will still receive advice on safe exercise practices but will not be eligible for randomisation and will be withdrawn from the study.

6.2. Inclusion Criteria

1. Participants must be able to give informed consent
2. Participants must be ≥65 years of age
3. Participants must have CKD stage 4 or 5
4. Participants are assessed as vulnerable or frail using the Clinical Frailty Scale (score ≥ 4)

6.3. Exclusion Criteria

1. Unstable Angina or recent (within the last 3 months) myocardial infarction
2. Recent (within the last 3 months) stroke or transient ischaemic attacks
3. Uncontrolled arrhythmias
4. Persistent uncontrolled hypertension (systolic blood pressure >180 or diastolic blood pressure >110)
5. Registered blind
6. Unable to mobilise independently
7. Receiving palliative care
8. Recently (within the last 12 months) enrolled in a structured exercise programme (e.g. cardiac rehabilitation programme) prescribed by a health professional
9. Anticipated to commence dialysis or receive a renal transplant within the next 3 months
10. Insufficient understanding of English language to complete study questionnaires or follow advice within the EX-FRAIL CKD Trial Exercise Guidebook.

6.4. Withdrawal Criteria

1. Participant is assessed as robust using the Frailty Phenotype criteria.
2. Participant has a new diagnosis of any of the following:
    a. Unstable Angina or myocardial infarction
    b. Stroke or transient ischaemic attack
    c. Persistent uncontrolled arrhythmia
    d. Persistent uncontrolled hypertension (systolic blood pressure >180 or diastolic blood pressure >110)
3. Participant suffers a fall sustaining a bony injury
4. Participant has a surgical operation

7. RANDOMISED INTERVENTION

7.1. Exercise Programme

The exercise programme is based upon published studies that have examined the use of exercise in patients with CKD and studies that have investigated the benefits of exercise in frail, older patients. (10, 12, 15, 16, 19-25) The exercises within the exercise programme aim to improve and maintain functional independence, through developing balance, coordination and lower limb strength.

Each exercise has 4 levels of difficulty. Participants meeting the FP frailty criteria will commence each exercise at level 1. Participants meeting the FP pre-frailty criteria will commence each exercise at level 2.

Participants will be asked to aim for three exercise sessions at home per week with each session lasting approximately 30-45 minutes.

Participants should aim to perform each exercise at a moderate intensity (Borg Scale Score between 12-16). If participants can perform an exercise comfortably (Borg Scale Score <12), participants will be asked to progress to the next level for that exercise.(26, 27)

Participants will be asked to record each exercise session in a personal exercise diary.

Participants will be telephoned on a weekly basis to monitor progress (including adherence and adverse events) and to provide ongoing guidance and support.

Exercise 1: Walking (warm-up; aerobic exercise)

Use your normal walking aid (this can be indoors or outdoors depending on where you would usually walk).

<u>Level 1</u>

Instructions: Walk for 1 minute.

<u>Level 2</u>

Instructions: Walk for 5 mins.

<u>Level 3</u>

Instructions: Walk for 10 mins.

<u>Level 4</u>

Instructions: Walk for 15 mins.

Attempt the next exercise when you feel ready.

Exercise 2: Lower Leg Extension (lower limb strengthening)

Level 1

Starting Position: Sitting upright in a chair.

Instructions: Slide your right foot forward as far as you can and the back to the starting position. Repeat with your left foot. Repeat 10 times.

Level 2

Starting position: Sitting on an upright chair.

Instructions: Straighten your right knee pulling your toes up towards you at the same time. Hold for 3 seconds and lower slowly. Repeat with your left knee. Repeat 10 times.

Level 3

Starting position: Sitting on an upright chair.

Instructions: As for level 2 with a 0.5 kg weight around your ankle. Repeat 10 times.

Level 4

Starting position: Sitting on an upright chair.

Instructions: As for level 2 with a 1 kg weight around your ankle. Repeat 10 times.

Perform 3 sets. Rest between sets, only attempting the next set when you feel ready.

After completing 3 sets, move on to the next exercise when you feel ready.

Exercise 3: Bilateral Calf Raises (lower limb strengthening; balance)

<u>Level 1</u>

Starting position: Sitting on an upright chair.

Instructions: Lift both heels off the floor keeping weight through your big toe and second toe. Hold for 3 seconds and lower slowly. Repeat 10 times.

<u>Level 2</u>

Starting position: Standing at a table or work surface and holding on with both hands.

Instructions: Lift both heels off the floor keeping weight through your big toe and second toe. Hold for 3 seconds and lower slowly. Repeat 10 times.

<u>Level 3</u>

Starting position: Standing at a table or work surface and place your finger tips on the surface for balance.

Instructions: Lift both heels off the floor keeping weight through your big toe and second toe. Hold for 3 seconds and lower slowly. Repeat 10 times.

<u>Level 4</u>

Starting position: Standing at a table or work surface without holding on.

Instructions: Lift both heels off the floor keeping weight through your big toe and second toe. Hold for 3 seconds and lower slowly. Repeat 10 times.

Perform 3 sets. Rest between sets, only attempting the next set when you feel ready.

After completing 3 sets, move on to the next exercise when you feel ready.

Exercise 4: Sit to Stand (lower limb strengthening; balance)

Level 1

Starting position: Sitting on an upright chair.

Instructions: Stand up and then sit down again using your arms to help you. Repeat 10 times. Rest between repetitions if you need to.

Level 2

Starting position: Sitting on an upright chair.

Instructions: Stand up and then sit down again without using your arms to help you. Repeat 10 times. Rest between repetitions if you need to.

Level 3

Starting position: Sitting on an upright chair.

Instructions: Stand up and then sit down again holding a 0.5kg weight in both hands. Do not wrap the weight around your wrists, especially if you have a fistula. Repeat 10 times. Rest between repetitions if you need to.

Level 4

Starting position: Sitting on an upright chair.

Instructions: Stand up and then sit down again holding a 1kg weight in both hands. Do not wrap the weight around your wrists, especially if you have a fistula. Repeat 10 times. Rest between repetitions if you need to.

Perform 3 sets. Rest between sets, only attempting the next set when you feel ready.

After completing 3 sets, move on to the next exercise when you feel ready.

Exercise 5: Wall/Chair Push Ups (upper limb strengthening)

<u>Level 1</u>
Starting position: Stand facing a wall.
Instructions: Put both palms on the wall about shoulder height. Lean forward, bending at the elbows, until your nose almost touches the wall. Push slowly away from the wall with your arms until you are standing straight up. Repeat 5 times. Rest between repetitions if you need to.

<u>Level 2</u>
Starting position: Stand facing a wall.
Instructions: Put both palms on the wall about shoulder height. Lean forward, bending at the elbows, until your nose almost touches the wall. Push slowly away from the wall with your arms until you are standing straight up. Repeat 10 times. Rest between repetitions if you need to.

<u>Level 3</u>
Starting position: Facing a sturdy chair.
Instructions: Lower yourself forward into the chair by bending at the elbows. Lower your body as far as you can, keeping your back and knees straight. Push slowly back up with your arms to the leaning position. Repeat 5 times. Rest between repetitions if you need to.

<u>Level 4</u>
Starting position: Facing a sturdy chair.
Instructions: Lower yourself forward into the chair by bending at the elbows. Lower your body as far as you can, keeping your back and knees straight. Push slowly back up with your arms to the leaning position. Repeat 10 times. Rest between repetitions if you need to.

Perform 3 sets. Rest between sets, only attempting the next set when you feel ready.

After completing 3 sets, move on to the next exercise when you feel ready.

Exercise 6: Marching/Stair Step (lower limb strengthening; balance)

Level 1

Starting position: Sitting on an upright chair

Instructions: Lift your right leg then your left leg (as though you are marching on the spot) 10 times.

Level 2

Starting position: Standing at a table or work surface and holding on with both hands.

Instructions: Lift your right leg and then your left leg (as though you are marching on the spot) 10 times.

Level 3

Starting position: Standing at a table or work surface without holding on.

Instructions: Lift your right leg and then your left leg (as though you are marching on the spot) 10 times.

Level 4

Starting Position: Stand up straight and face a small step.

Instructions: Balance with a railing or on a wall. Step up onto the step with your right foot and then your left. Next, step back down, first with your right foot, then your left. Alternate legs. Repeat 10 times.

Perform 3 sets. Rest between sets, only attempting the next set when you feel ready.

7.2. Risk Assessment and Safety Monitoring

We recognise that frail older adults with CKD are a vulnerable population. Furthermore, we appreciate that we are asking that some participants perform exercises independently and unsupervised. Exercise programmes have been previously performed safely by those with pre-dialysis CKD and dialysis-dependent CKD.(15, 16) Furthermore, studies have safely been performed that evaluate the use of exercise programmes for frail, older adults.(10, 11, 28, 29) Studies have also safely used home-based exercise programmes.(25) Thus, these studies have demonstrated that exercise in frail populations is possible and safe, when the appropriate precautions are followed. Consequently, we have taken several measures to minimise the risks to participants in the exercise group. The eligibility criteria are extensive so that high risk patients are not recruited to this feasibility study. This includes patients with unstable medical conditions. Each potential participant will be assessed by a medical practitioner to ensure their suitability.

Participants will be telephoned on a weekly basis. If the participant has experienced an adverse event, a medical practitioner will contact the patient and arrange assessment as clinically appropriate.

Participants will be advised not to exercise in the following circumstances:

- If they are feeling unwell or feverish
- If they have developed a new illness or symptom since the last exercise session
- If exercising causes pain

If any of the above occur, participants will be advised to seek medical attention, either by contacting their local GP or, in more urgent cases, by telephoning 111. Participants will also be asked to contact a clinical member of the research team before they exercise again.

Participants will be advised that they should stop exercising immediately in the following circumstances:

- If they are uncomfortably short of breath

- If they develop pain or discomfort in chest, neck or jaw
- If they experience palpitations
- If they feel dizzy or lightheaded
- If they feel nauseated
- If they suffer cramps
- If they experience visual disturbance
- If they feel excessively fatigued i.e. struggling to perform everyday activities that they would otherwise perform comfortably

Participants will be asked to seek urgent medical attention by telephoning 111 or 999 if they develop new symptoms that persist despite stopping exercise and in all cases of chest, jaw and neck pain/discomfort. Participants will be asked to contact a member of the research team in the above circumstances before they start exercising again.

8. STUDY PROCEDURES AND ASSESSMENTS

8.1. Time and Events table

**Procedures and Assessments**	**Week**													
	0	1	2	3	4	5	6	7	8	9	10	11	12	13-16
Record demographics and medical history	X												X	
Height, weight, BP and HR	X												X	
Semi-structured interviews														X
FP Assessment<br>Review weight changes<br>Measure grip strength<br>Gait Speed (15 feet)<br>Physical activity questionnaire<br>Exhaustion assessment	X												X	
SPPB<br>Sit to stand x 5<br>Balance testing<br>Gait speed (8 feet)	X												X	
DASI	X												X	
SF-36	X												X	
FES-I	X												X	
POS-S RENAL	X												X	
Exercise programme education<br>(only those allocated to exercise)	X													
Telephone call<br>(only those allocated to exercise)		X	X	X	X	X	X	X	X	X	X	X		
Diary Entries<br>(only those allocated to exercise)		X	X	X	X	X	X	X	X	X	X	X	X	

Table 1. Schedule of Assessments

8.2. Participant Acceptability and Adherence

Semi-structured, individual interviews will be performed with a purposively selected group of participants from both groups. A representative sample (based on age, gender and frailty status) of participants will be asked a series of open-ended questions relating to their experience of the trial and, where applicable, the intervention. The questions will broadly assess the following parameters:

- Overall impressions of the trial and the exercise programme
- Acceptability of randomisation, study outcome measures and the exercise programme
- Adherence with the exercise programme
- Perceived safety of the exercise programme
- Any other participant initiated reflections or observations

Participants may bring along a friend or relative, if they wish. The interviews will be arranged at the participant's convenience and take place in a comfortable, private room at the hospital. Interviews will be recorded using a digital recorder with the participant's consent. At the start of the interview the researcher will introduce the session, explaining the background and reasons for the study, how the interview will proceed and the details of audio-recording and note-taking. Ground rules for the interview will be set (e.g. keeping the interview anonymous and confidential) and participants will have the opportunity to ask questions. Once the participant is ready, the interview will begin. At this point, the audio-recording will start and the researcher may make notes on what is said. The interview will last for no more than 60 minutes and can be stopped at any point if the participant wishes, for example if they become fatigued. Participants will not be asked to discuss anything they don't feel comfortable to talk about. The audio-recording will be transcribed and quotations finalised by Dr Nixon. All quotations will be anonymised.

Participants within the exercise group will be asked to record each exercise session in a personal exercise diary.

8.3. Fried Physical Frailty Phenotype

The FP criteria has been extensively used in CKD to assess frailty.(6, 7, 18, 30) Using the FP Criteria we will define physical frailty as the presence of at least three of the following:

- Unintentional weight loss defined as a loss of 10 pounds/4.5kg (or ≥ 5% of body weight) over the preceding 12 months.(18)
- Weakness defined as grip strength in the lowest 20th percentile of the Cardiovascular Health Study Score (adjusted for gender and body mass index).(18)
- Slow walking speed defined as time to walk 15 feet in the lowest 20th percentile of the Cardiovascular Health Study.(18)
- Low physical activity defined for men as < 383 Kcals of physical activity per week and for women <270 Kcals of physical activity per week.(18, 31) Physical activity will be assessed using a short version of the Minnesota Leisure Time Activity Questionnaire.
- Exhaustion defined as answering '2' or '3' to either of the following questions based upon the CES-D Depression Scale statement:
    - How often did you feel that everything you did was an effort?
        - 0 = rarely or none of the time (<1 day).
        - 1 = some or a little of the time (1-2 days).
        - 2 =moderate amount of the time (3-4 days).
        - 3 = most of the time.(18, 32)
    - How often did you feel like you could not get going?
        - 0 = rarely or none of the time (<1 day).
        - 1 = some or a little of the time (1-2 days).
        - 2 =moderate amount of the time (3-4 days).
        - 3 = most of the time.(18, 32)

8.4. Lower extremity physical function

The Short Physical Performance Battery (SPPB) is a rapidly administered test to measure physical function.(30, 33, 34)

The SPPB is a group of measures that combine to give a composite score:

- Repeated Chair Stands: participants will be asked to stand from a seated position five times in succession. The time to complete this will be recorded and used to calculate a chair stand ordinal score.
- Balance testing: participants will be asked to stand in three positions with increasing difficulty. If they are unable to perform a position, they will not progress to the next position. The positions include standing with feet side by side, a semi-tandem stand (heel of one foot placed by the big toe of the other foot) and full tandem position. Participants will be timed in these positions (maximum 10 seconds) and ordinal scoring completed.
- Gait Speed Testing: participants will be timed walking 8 feet (2.44meters) at their usual walking speed. Gait ordinal scoring will be calculated based upon the participant's time.

8.5. Self-reported functional capacity

The Duke Activity Status Index (DASI) is short questionnaire used to assess exercise capacity.(35) The DASI questionnaire has been used in the CKD population.(25, 36)

8.6. Health-related quality of life

The Medical Outcomes Study Short Form-36 (SF-36) will be used to assess quality of life.(37) The SF-36 has been extensively used in studies involving CKD patients.(38-41)

8.7. Fear of falling

Participants will be asked to complete the Falls Efficacy Scale-International (FES-I) tool at the baseline study visit and at the end of the study period.(42-44)

8.8. Symptom Burden

Participants will be asked to complete the Palliative Care Outcome Scale- Symptoms RENAL (POS-S RENAL) at the baseline study visit and at the end of the study period.(45, 46)

9. DATA MANAGEMENT

Data will be recorded on Case Report Forms and stored in a secure room in the NIHR Lancashire Clinical Research Facility at LTHTR.

Participant data will then be recorded on an electronic database on the LTHTR server. All electronic data will be password protected. Confidentiality of personal data will be maintained throughout the study. There will be pseudoanonymisation of data only whilst necessary, after which there will be complete anonymisation of data.

Management of clinical data will be performed in accordance with the NHS Code of Confidentiality and the LTHTR Information Governance Policy.

10. STATISTICAL CONSIDERATIONS AND DATA ANALYSES

In accordance with published recommendations, we will recruit 60 participants (with a participant ratio 1:1) to assess the feasibility of a larger definitive randomised controlled trial.(47) We anticipate that approximately 18 participants should be sufficient to provide a representative sample for the qualitative sub-study, with a participant ratio 2:1 in favour of those allocated to the exercise programme.

Descriptive statistics will be used to summarise demographic data and clinical variables. Categorical data will be reported using frequencies and percentages. Quantitative variables will be reported using either the mean and standard deviation or the median and interquartile range depending on the distribution of the data. From the information gathered in this study, the sample size needed for a definitive randomised controlled trial will be calculated.

The transcribed interviews will be analysed using a flexible form of thematic analysis whereby narrative segments will first be coded and then translated into more abstract themes in an iterative manner.(48) Two of the researchers (Dr Nixon and Mr Finlayson) will compare and contrast the emerging themes to ensure all of the codes are represented. A more detailed explication of each theme will then be developed and used to inform the write-up of study findings.

11. PROGRESSION CRITERIA

We will use a red/amber/green traffic light (stop/amend/continue) approach to the study progression criteria as described in the literature.(49) Hence, if a progression criterion is not met, we will use data gathered during the study to revise the study protocol.

1. Recruitment

- Greater than 10% of patients attending General Nephrology Clinics eligible for FP Assessment
- Of those eligible for FP assessment, greater than 60% to be eligible for randomisation (i.e. pre-frail or frail as assessed by the FP).
- These values are based upon data from our current database of patient CFS scores and corresponding FP scores.
- Note: there are over 280 patients seen in General Nephrology Clinics each month at Royal Preston Hospital alone.

2. Exercise Programme Adherence

- Greater than 60% randomised to the exercise programme to complete at least two exercise sessions per week during the 12-week exercise programme.
- If this is not achieved, we will explore barriers raised by participants during interviews.

3. Outcome Data

- Greater than 70% of participants who adhere to the exercise programme to complete all outcome assessments.
- If this is not achieved, we will explore barriers raised by participants during interviews.

12. STUDY GOVERNANCE

12.1. Regulatory and Ethical Considerations

Prior to study initiation, a favourable ethical opinion will be obtained from the NHS Health Research Authority. All research activity will be conducted in accordance with the International Standard of Good Clinical Practice. The study sponsor will be LTHTR and all research activity will be conducted in accordance with trust policies. This feasibility study will be registered with the International Standard Randomised Controlled Trial Number Registry.

12.2. Study Monitoring

The LTHTR Internal Research Audit Programme ensures all research activities performed in the trust are done so in accordance with the Standards of Good Clinical Practice, UK Clinical Trials Legislation and Trust Policies. This study will be registered with the National Institute for Health Research (NIHR) and subject to their auditing programme.

12.3. Records Retention

Following completion of the study, study records will be maintained in a secure location at LTHTR for 15 years.

12.4. Study Steering Committee

The study steering committee provides scientific, ethical and financial oversight of all research activity. The committee comprises two clinicians, two academic researchers, two physiotherapists, one research nurse and one patient. Regular meetings will be held throughout the study. The patient member of the committee and will be reimbursed for travel costs and receive participation rewards as recommended by INVOLVE.

13. PUBLIC AND PATIENT INVOLVEMENT

The study has been presented to the LTHTR Research Development Group, which includes lay members. This has influenced the design of the study from a quantitative study to a study using a mixed methods approach.

The study has been presented at the Lancashire and South Cumbria Kidney Patient Association Annual General Meeting, which served as a forum to receive feedback from patients and the public. A lay summary of the proposed study has also been published in the Lancashire and South Cumbria Kidney Patient Association 2017 Christmas Newsletter.

The study has been developed with the support of the NIHR Research Design Service North West (RDS NW). RDS NW funding has supported a patient discussion group. Four patients and one patient's relative attended. Positive feedback was received on proposed study visits, assessments and exercises. Feedback from the group included:

- That as the exercise programme was simple, participants will be encouraged to complete the exercises.
- The exercise programme appears safe and feasible for frail participants with chronic kidney disease to complete at home.

A patient is a member of the study steering committee that provides scientific, ethical and financial oversight of all research activity. The study steering committee will meet regularly throughout the study. The patient member of the committee will be reimbursed for travel costs and receive participation rewards as recommended by INVOLVE.

Study updates will be provided to the Lancashire and South Cumbria Kidney Patient Association at their Annual General Meetings. This will provide a useful opportunity to receive regular feedback from patients and the public. In addition, all participants will be offered the opportunity to receive a lay-description summary of the study findings.

14. IMPACT SUMMARY

Participants within the EX-FRAIL CKD trial randomised to exercise will be provided expert guidance and support to increase their physical activity levels. Participants that have a sustained increase in activity levels may have an associated improvement in their health-related quality of life and survival.

We will present our findings at relevant conferences including the LTHTR Research and Innovation Showcase and to the local kidney charity, the Lancashire and South Cumbria Kidney Patient Association. We will publish our findings in relevant journal(s) to inform the wider academic community of our results. This hopefully will generate further discussion and research ideas on how to develop management strategies to improve the health and well-being of pre-frail and frail older people with CKD and other chronic health conditions.

The information gathered in the EX-FRAIL CKD trial will inform the design of a definitive randomised controlled trial that investigates the effects of a home-based exercise programme on the physical function and quality of life of pre-frail and frail older patients with CKD. If we demonstrate positive study findings, we hope to develop a study that investigates the long-term health outcomes of a multi-domain intervention, which incorporates the EX-FRAIL CKD home-based exercise programme, for pre-frail and frail older patients with CKD.(50) In addition, we plan to develop technology that will support frail, older patients with CKD participate in home-based exercise programmes, including the use of instructional web-based videos and supporting smart phone applications.

Positive study findings should also encourage policy makers to support the provision of regional home-based exercise services for pre-frail and frail older adults living with CKD. Such a service would likely be associated with increased functional independence of older adults living in the UK with chronic kidney disease, therefore reducing social care demand and falls-related hospital admissions. It may also increase the likelihood that an older individual with advanced CKD is considered eligible to receive a kidney transplant, as they

would be more likely to have a successful post-transplant outcome. This will reduce demand for costlier renal replacement therapies.

REFERENCES

1. Johansen KL, Chertow GM, Jin C, Kutner NG. Significance of frailty among dialysis patients. Journal of the American Society of Nephrology : JASN. 2007;18(11):2960-7.

2. Ballew SH, Chen Y, Daya NR, Godino JG, Windham BG, McAdams-DeMarco M, et al. Frailty, Kidney Function, and Polypharmacy: The Atherosclerosis Risk in Communities (ARIC) Study. American journal of kidney diseases : the official journal of the National Kidney Foundation. 2016.

3. Collard RM, Boter H, Schoevers RA, Oude Voshaar RC. Prevalence of frailty in community-dwelling older persons: a systematic review. J Am Geriatr Soc. 2012;60(8):1487-92.

4. Roshanravan B, Khatri M, Robinson-Cohen C, Levin G, Patel KV, de Boer IH, et al. A prospective study of frailty in nephrology-referred patients with CKD. American journal of kidney diseases : the official journal of the National Kidney Foundation. 2012;60(6):912-21.

5. Bao Y, Dalrymple L, Chertow GM, Kaysen GA, Johansen KL. Frailty, dialysis initiation, and mortality in end-stage renal disease. Archives of internal medicine. 2012;172(14):1071-7.

6. McAdams-DeMarco MA, Law A, Salter ML, Boyarsky B, Gimenez L, Jaar BG, et al. Frailty as a novel predictor of mortality and hospitalization in individuals of all ages undergoing hemodialysis. J Am Geriatr Soc. 2013;61(6):896-901.

7. McAdams-DeMarco MA, Suresh S, Law A, Salter ML, Gimenez LF, Jaar BG, et al. Frailty and falls among adult patients undergoing chronic hemodialysis: a prospective cohort study. BMC Nephrol. 2013;14:224.

8. Lee SJ, Son H, Shin SK. Influence of frailty on health-related quality of life in pre-dialysis patients with chronic kidney disease in Korea: a cross-sectional study. Health and quality of life outcomes. 2015;13:70.

9. Mansur HN, Colugnati FA, Grincenkov FR, Bastos MG. Frailty and quality of life: a cross-sectional study of Brazilian patients with pre-dialysis chronic kidney disease. Health and quality of life outcomes. 2014;12:27.

10. de Labra C, Guimaraes-Pinheiro C, Maseda A, Lorenzo T, Millan-Calenti JC. Effects of physical exercise interventions in frail older adults: a systematic review of randomized controlled trials. BMC Geriatr. 2015;15:154.

11. Theou O, Stathokostas L, Roland KP, Jakobi JM, Patterson C, Vandervoort AA, et al. The effectiveness of exercise interventions for the management of frailty: a systematic review. Journal of aging research. 2011;2011:569194.

12. Clegg AP, Barber SE, Young JB, Forster A, Iliffe SJ. Do home-based exercise interventions improve outcomes for frail older people? Findings from a systematic review. Rev Clin Gerontol. 2012;22(1):68-78.

13. Kim JC, Kalantar-Zadeh K, Kopple JD. Frailty and protein-energy wasting in elderly patients with end stage kidney disease. Journal of the American Society of Nephrology : JASN. 2013;24(3):337-51.

14. John SG, Sigrist MK, Taal MW, McIntyre CW. Natural history of skeletal muscle mass changes in chronic kidney disease stage 4 and 5 patients: an observational study. PloS one. 2013;8(5):e65372.

15. Heiwe S, Jacobson SH. Exercise training for adults with chronic kidney disease. The Cochrane database of systematic reviews. 2011(10):Cd003236.

16. Heiwe S, Jacobson SH. Exercise training in adults with CKD: a systematic review and meta-analysis. American journal of kidney diseases : the official journal of the National Kidney Foundation. 2014;64(3):383-93.

17. Rockwood K, Song X, MacKnight C, Bergman H, Hogan DB, McDowell I, et al. A global clinical measure of fitness and frailty in elderly people. CMAJ : Canadian Medical Association journal = journal de l'Association medicale canadienne. 2005;173(5):489-95.

18. Fried LP, Tangen CM, Walston J, Newman AB, Hirsch C, Gottdiener J, et al. Frailty in older adults: evidence for a phenotype. J Gerontol A Biol Sci Med Sci. 2001;56(3):M146-56.

19. Painter P. Exercise: A Guide for the People on Dialysis: The Life Options Rehabilitation Advisory Council; 2000 [Available from: http://old.lifeoptions.org/catalog/pdfs/booklets/exercise.pdf.

20. Land S, Dinan S. Preventing falls: Strength and balance exercises for healthy ageing: Age UK.; [Available from: http://www.ageuk.org.uk/Documents/EN-GB/strength_and_balance_training_PDF.pdf?dtrk=true.

21. Rossi AP, Burris DD, Lucas FL, Crocker GA, Wasserman JC. Effects of a renal rehabilitation exercise program in patients with CKD: a randomized, controlled trial. Clin J Am Soc Nephrol. 2014;9(12):2052-8.

22. Koufaki P, Greenwood S, Painter P, Mercer T. The BASES expert statement on exercise therapy for people with chronic kidney disease. Journal of sports sciences. 2015;33(18):1902-7.

23. Clegg A, Barber S, Young J, Iliffe S, Forster A. The Home-based Older People's Exercise (HOPE) trial: a pilot randomised controlled trial of a home-based exercise intervention for older people with frailty. Age Ageing. 2014;43(5):687-95.

24. Cesari M, Vellas B, Hsu FC, Newman AB, Doss H, King AC, et al. A physical activity intervention to treat the frailty syndrome in older persons-results from the LIFE-P study. J Gerontol A Biol Sci Med Sci. 2015;70(2):216-22.

25. Greenwood SA, Lindup H, Taylor K, Koufaki P, Rush R, Macdougall IC, et al. Evaluation of a pragmatic exercise rehabilitation programme in chronic kidney disease. Nephrol Dial Transplant. 2012;27 Suppl 3:iii126-34.

26. Borg G. Perceived exertion as an indicator of somatic stress. Scandinavian journal of rehabilitation medicine. 1970;2(2):92-8.

27. Borg G. Psychophysical scaling with applications in physical work and the perception of exertion. Scandinavian Journal of Work, Environment & Health. 1990;16:55-8.

28. Tarazona-Santabalbina FJ, Gomez-Cabrera MC, Perez-Ros P, Martinez-Arnau FM, Cabo H, Tsaparas K, et al. A Multicomponent Exercise Intervention that Reverses Frailty and Improves Cognition, Emotion, and Social Networking in the Community-Dwelling Frail Elderly: A Randomized Clinical Trial. Journal of the American Medical Directors Association. 2016;17(5):426-33.

29. Serra-Prat M, Sist X, Domenich R, Jurado L, Saiz A, Roces A, et al. Effectiveness of an intervention to prevent frailty in pre-frail community-dwelling older people consulting in primary care: a randomised controlled trial. Age Ageing. 2017.

30. Bohm C, Storsley L, Tangri N. The assessment of frailty in older people with chronic kidney disease. Curr Opin Nephrol Hypertens. 2015;24(6):498-504.

31. Taylor HL, Jacobs DR, Jr., Schucker B, Knudsen J, Leon AS, Debacker G. A questionnaire for the assessment of leisure time physical activities. Journal of chronic diseases. 1978;31(12):741-55.

32. Orme JG, Reis J, Herz EJ. Factorial and discriminant validity of the Center for Epidemiological Studies Depression (CES-D) scale. Journal of clinical psychology. 1986;42(1):28-33.

33. Guralnik JM, Simonsick EM, Ferrucci L, Glynn RJ, Berkman LF, Blazer DG, et al. A short physical performance battery assessing lower extremity function: association with self-reported disability and prediction of mortality and nursing home admission. Journal of gerontology. 1994;49(2):M85-94.

34. Reese PP, Cappola AR, Shults J, Townsend RR, Gadegbeku CA, Anderson C, et al. Physical performance and frailty in chronic kidney disease. Am J Nephrol. 2013;38(4):307-15.

35. Hlatky MA, Boineau RE, Higginbotham MB, Lee KL, Mark DB, Califf RM, et al. A brief self-administered questionnaire to determine functional capacity (the Duke Activity Status Index). The American journal of cardiology. 1989;64(10):651-4.

36. Ravani P, Kilb B, Bedi H, Groeneveld S, Yilmaz S, Mustata S. The Duke Activity Status Index in patients with chronic kidney disease: a reliability study. Clin J Am Soc Nephrol. 2012;7(4):573-80.

37. Hays RD, Sherbourne CD, Mazel R. User's Manual for the Medical Outcomes Study (MOS) Core Measures of Health-Related Quality of Life 1995 [Available from: https://www.rand.org/pubs/monograph_reports/MR162.html.

38. Mitema D, Jaar BG. How Can We Improve the Quality of Life of Dialysis Patients? Seminars in dialysis. 2016;29(2):93-102.

39. Lowrie EG, Curtin RB, LePain N, Schatell D. Medical outcomes study short form-36: a consistent and powerful predictor of morbidity and mortality in dialysis patients. American journal of kidney diseases : the official journal of the National Kidney Foundation. 2003;41(6):1286-92.

40. DeOreo PB. Hemodialysis patient-assessed functional health status predicts continued survival, hospitalization, and dialysis-attendance compliance. American journal of kidney diseases : the official journal of the National Kidney Foundation. 1997;30(2):204-12.

41. Kalantar-Zadeh K, Kopple JD, Block G, Humphreys MH. Association among SF36 quality of life measures and nutrition, hospitalization, and mortality in hemodialysis. Journal of the American Society of Nephrology : JASN. 2001;12(12):2797-806.

42. Kempen GI, Yardley L, van Haastregt JC, Zijlstra GA, Beyer N, Hauer K, et al. The Short FES-I: a shortened version of the falls efficacy scale-international to assess fear of falling. Age Ageing. 2008;37(1):45-50.
43. Yardley L, Beyer N, Hauer K, Kempen G, Piot-Ziegler C, Todd C. Development and initial validation of the Falls Efficacy Scale-International (FES-I). Age Ageing. 2005;34(6):614-9.
44. Delbaere K, Close JC, Mikolaizak AS, Sachdev PS, Brodaty H, Lord SR. The Falls Efficacy Scale International (FES-I). A comprehensive longitudinal validation study. Age Ageing. 2010;39(2):210-6.
45. Brown MA, Collett GK, Josland EA, Foote C, Li Q, Brennan FP. CKD in elderly patients managed without dialysis: survival, symptoms, and quality of life. Clin J Am Soc Nephrol. 2015;10(2):260-8.
46. Murphy EL, Murtagh FE, Carey I, Sheerin NS. Understanding symptoms in patients with advanced chronic kidney disease managed without dialysis: use of a short patient-completed assessment tool. Nephron Clin Pract. 2009;111(1):c74-80.
47. Lancaster GA, Dodd S, Williamson PR. Design and analysis of pilot studies: recommendations for good practice. Journal of evaluation in clinical practice. 2004;10(2):307-12.
48. Braun V, Clarke V. Using thematic analysis in psychology. Qualitative Research in Psychology. 2006;3(2):77-101.
49. Avery KN, Williamson PR, Gamble C, O'Connell Francischetto E, Metcalfe C, Davidson P, et al. Informing efficient randomised controlled trials: exploration of challenges in developing progression criteria for internal pilot studies. BMJ open. 2017;7(2):e013537.
50. Dedeyne L, Deschodt M, Verschueren S, Tournoy J, Gielen E. Effects of multi-domain interventions in (pre)frail elderly on frailty, functional, and cognitive status: a systematic review. Clin Interv Aging. 2017;12:873-96.
