# Supplementary material for: Home-based exercise for people living with frailty and chronic kidney disease: A mixed-methods pilot randomised controlled trial
Source: PLoS One. 2021 Jul 1;16(7):e0251652. doi: 10.1371/journal.pone.0251652 (PMC8248609; doi:10.1371/journal.pone.0251652)
Supplement: Attachment — Submitted filename: Response Letter_April.docx [file pone.0251652.s007.docx]

Department of Renal Medicine

Royal Preston Hospital

Sharoe Green Lane

Preston

United Kingdom

10^th^ April 2020

Dear Editorial Board,

We thank the Editors and Reviewers for taking the time to consider our manuscript **‘Home-based exercise for people living with frailty and chronic kidney disease: a mixed-methods pilot randomised controlled trial’** for publication as a clinical trial in PLOS One. Thank you also for the extension to the time usually permitted for a response. We endeavour to respond to the Academic Editor’s and Reviewers’ points comprehensively below.

**Academic Editor**

1. We have now formatted the manuscript to meet PLOS ONE’s style requirements.
2. We have provided additional information about the eligibility criteria and about the intervention.
3. We have provided additional information on qualitative methodology.
4. We have updated our Competing Interests section.
5. An abstract of preliminary results was presented at an ERA-EDTA conference and the abstract was published in a NDT supplementary issue, associated with the ERA-EDTA conference. The manuscript under consideration for publication in PLOS ONE, however, contains the full details of the study and updated results that have not been published previously.
6. The minimal anonymized data set necessary to replicate study findings is now available at <https://doi.org/10.5061/dryad.3ffbg79hp>.

**Reviewer 1**

1. We are uncertain as to what is meant by the statistical methods used to calculate the 95% confidence intervals. All 95% confidence intervals were calculated using the described statistical software, e.g. 95% confidence intervals of proportions using R (version 4.0.2, R Foundation for Statistical Computing). We would be happy to revise again if the Reviewer could provide further detail on this point.
2. Thank you for this comment. We have updated the section titled ‘Sample Size Estimation’ to improve clarity.

**Reviewer 2**

1. Thank you for highlighting these papers. We have amended the Introduction to acknowledge the systematic review and meta-analysis, which includes all the mentioned papers.
2. Thank you for highlighting this. We acknowledge that exercise diaries and telephone calls are subject to recall bias, though do not entirely agree that the use of exercise diaries and telephone calls is a *major* limitation of the study. We have amended the limitations section of the Discussion to address this point.
3. Although participants had a high Barthel Index score, they were assessed as pre-frail or frail using the Frailty Phenotype. There is overlap between disability and frailty, but an individual can be frail without having disability; frailty itself can affect ability to participate in physical activity. Moreover, The American College of Sports Medicine and American Heart Association Guidelines highlight the importance of physical activity being increased gradually in older adults, particularly those that are very deconditioned. Participants used the Borg Rating of Perceived Exertion (RPE) to report their perceived exertion during each exercise session; these scores were used to guide the progression to more challenging exercise levels each week. The intended RPE exercises 2-6 was achieved (mean RPE score for exercises 2-6 was 12±2). Considering the above, we do not believe that the intensity of the exercise programme affected the trial feasibility outcomes.
4. Barthel Index scores are now presented as median and interquartile range. The results are presented in the Results section titled ‘Physical Function and Patient Reported Outcome Measures’.

Thank you for considering our revised manuscript. We hope that you are satisfied with our responses and we look forward to hearing from you.

Yours sincerely,

Dr Andrew Nixon

Renal Medicine and General Internal Medicine Specialist Trainee
